# Development of TaqMan Real-Time PCR Protocols for Simultaneous Detection and Quantification of the Bacterial Pathogen *Ralstonia solanacearum* and Their Specific Lytic Bacteriophages

**DOI:** 10.3390/v15040841

**Published:** 2023-03-25

**Authors:** Edson Bertolini, Àngela Figàs-Segura, Belén Álvarez, Elena G. Biosca

**Affiliations:** 1 Departamento de Microbiología y Ecología, Universitat de València (UV), 46100 Valencia, Spain; 2 Faculdade de Agronomia, Universidade Federal do Rio Grande do Sul (UFRGS), Porto Alegre 91540-000, Brazil; 3 Departamento de Investigación Aplicada y Extensión Agraria, Instituto Madrileño de Investigación y Desarrollo Rural, Agrario y Alimentario (IMIDRA), 28805 Alcalá de Henares, Spain

**Keywords:** bacterial wilt, phage, identification, enumeration, duplex, multiplex

## Abstract

*Ralstonia solanacearum* is the causal agent of bacterial wilt, one of the most destructive diseases of solanaceous plants, affecting staple crops worldwide. The bacterium survives in water, soil, and other reservoirs, and is difficult to control. In this sense, the use of three specific lytic *R. solanacearum* bacteriophages was recently patented for bacterial wilt biocontrol in environmental water and in plants. To optimize their applications, the phages and the bacterium need to be accurately monitored and quantified, which is laborious and time-consuming with biological methods. In this work, primers and TaqMan probes were designed, and duplex and multiplex real-time quantitative PCR (qPCR) protocols were developed and optimized for the simultaneous quantification of *R. solanacearum* and their phages. The quantification range was established from 10^8^ to 10 PFU/mL for the phages and from 10^8^ to 10^2^ CFU/mL for *R. solanacearum*. Additionally, the multiplex qPCR protocol was validated for the detection and quantification of the phages with a limit ranging from 10^2^ targets/mL in water and plant extracts to 10^3^ targets/g in soil, and the target bacterium with a limit ranging from 10^3^ targets/mL in water and plant extracts to 10^4^ targets/g in soil, using direct methods of sample preparation.

## 1. Introduction

One of the most significant plant pathogens on a global scale is the bacterial wilt species *Ralstonia solanacearum* [1,2], as it produces disease in solanaceous crops basic for human nutrition such as potato and tomato, and ornamentals such as geranium [3,4,5]. The pathogen is soil- and water-borne, enters the plant through the roots, and infects vascular tissues, producing brown rot in potato tubers and lethal wilting in the plant [3,6,7]. Infection is related to many factors; among them, an unusually high number of virulence and pathogenicity determinants [8,9,10,11]. *R. solanacearum* is originally from tropical and subtropical areas but it can also survive in temperate climates. It is a quarantine bacterium in the European Union (EU), the USA, and Canada, and a Select Agent in the USA [12,13,14]. Following epidemic outbreaks, *R. solanacearum* can persist for variable periods in watercourses, soils, and other reservoirs in the absence of the host [3,4,15,16,17,18], and despite the exposure to environmental stress, without losing virulence [17,18]. The endurance of the bacterium points out potential for dissemination and colonization of new geographical and climatic areas. In environmental water, *R. solanacearum* cells resuscitated after just a favorable temperature upshift [18], which makes spread of the pathogen an ever-increasing threat under global warming conditions.

The presence of *R. solanacearum* in the environment constitutes a risk to sensitive crops, most of which are irrigated. In fact, the origin of many bacterial wilt outbreaks has been linked to contact with *R. solanacearum*-contaminated water [16,18,19], which is one of the main pathways for the introduction of the pathogen to new areas. The great ability for survival of *R. solanacearum* in aquatic habitats and the fact that water is an increasingly scarce essential resource [20], together with the lack of effective and/or environmentally friendly chemical control methods either in water or in the field, constitute a problem for farmers and agricultural production of susceptible crops in affected areas. To overcome this issue, and in agreement with the growing social awareness for a healthy environment and a demand for safe food, the interest has been focused on alternative bacterial wilt biological control methods [21,22]. In this sense, the use of lytic bacteriophages (phages) has the advantage of specifically targeting the bacterial host cell, allowing for maintenance of sustainable agroecosystems. In recent years, three phages with specific lytic activity against *R. solanacearum* were isolated from environmental water from different locations in Spain. They were characterized, named vRsoP-WF2, vRsoP-WM2, and vRsoP-WR2, and their efficacy to biocontrol the pathogen populations in environmental water and *in planta* was demonstrated for all of them and their combinations in laboratory assays, and patented [23,24,25,26,27]. The three phages constituted the first European phages isolated against *R. solanacearum*, and their genomic characterization allowed their classification in a novel species of the genus *Gyeongsanvirus* within the *Autographiviridae* family, as well as confirmation of their suitability as safe biocontrol agents [28]. Although first steps were taken towards their conservation with a view to their commercialization [27], there are still some unknown data concerning their applications in the field, especially when a mixture of the phages is used, which require the precise monitoring and quantification of the three phages and the target bacterium.

To date, the time course of phage–host dynamics in liquid medium is most frequently monitored based on optical density measurements of the loss of turbidity from an initial bacterial host suspension due to the lytic activity of a phage or a mixture of phages. To link these optical density data to quantification of host and phage populations, parallel counting on solid medium is usually performed by both direct plating and a standard double-layer plating, respectively, for the host bacterium and the phage or phages, at each sampling time [29,30,31,32]. This is laborious and time-consuming, and the efficiency mainly depends on several factors, such as the type of plaque, the target bacterium, and the culture medium [31]. Some other inconveniences consist of the difficulty to quantify bacterial hosts and phages in very short periods, especially when different experimental conditions are running simultaneously, and the inability to quantitatively differentiate each phage in a mixture, as well as host and phages in non-sterile conditions, such as environmental water, soil suspensions, or plant extracts. In contrast, real-time quantitative PCR (qPCR) has proven to be an accurate, reproducible, rapid, simple, and useful alternative, allowing for individual quantification of multiple bacteria and phages in mixed cultures [31,32,33,34]. With respect to *R. solanacearum*, several real-time qPCR protocols have been described [35,36,37,38,39,40,41]. However, no real-time qPCR protocol has been developed so far for phages with specific lytic activity against *R. solanacearum*. In this regard, in the present work, specific primers and TaqMan probes have been designed to develop two real-time qPCR protocols: a duplex qPCR for the simultaneous detection and quantification of the three patented *R. solanacearum* phages, vRsoP-WF2, vRsoP-WM2, and vRsoP-WR2, and the target bacterium, and a multiplex qPCR for detection of the three phages, the individual vRsoP-WR2 phage, and the target bacterium. Additionally, the multiplex real-time qPCR protocol has been validated for the detection and quantification of the three *R. solanacearum* phages and the target bacterium in water, soil, and plant material, mimicking samples taken under field conditions.

## 2. Materials and Methods

### 2.1. Bacterium, Phages, and Growth Conditions

The strain CFBP 4944 of *R. solanacearum* isolated from potatoes in Spain [19,26] was the bacterial host in all phage assays. It was cultured on the general media casamino acids peptone glucose (CPG) [42] or Luria Bertani (LB) [43], with 1.5% bacteriological agar (CPGA or LBA), for 48 h at 28 °C. LB broth (LBB) was used for overnight cultures of the bacterial strain at 28 °C with aeration by shaking at 120 rpm. *R. solanacearum* phages vRsoP-WF2, vRsoP-WM2, and vRsoP-WR2, further characterized by Biosca et al. [28], were used in this work. They were propagated on *R. solanacearum* cultures similarly to Álvarez et al. [26], with minor modifications. Briefly, the host strain was grown in LBB overnight at 28 °C and 120 rpm. Thereafter, the bacterial cell concentration was adjusted to an OD_600 nm_ = 0.5, equivalent to about 10^8^ colony-forming units (CFU)/mL, using a spectrophotometer (Thermo Scientific Genesys 20, Waltham, MA, USA). Afterwards, 0.1 mL of 0.22 μm-filtered lysates were added to 5 mL of adjusted bacterial suspensions and the mixture was incubated overnight at 28 °C with shaking at 120 rpm. Phage titers were determined according to [27] by performing serial 10-fold dilutions of each phage lysate (0.22 μm-filtered lysates) in SM buffer [44] and the double-layer agar method. Thus, 0.2 mL aliquots of the bacterial culture (adjusted to an OD_600 nm_ = 0.5) were mixed with 0.1 mL of each phage dilution and 5 mL of soft agar (0.6%) medium, poured onto CPGA plates, and incubated at 28 °C for 48 h. After incubation, the plaque-forming units (PFU) were observed. Phage suspensions were maintained for short periods at 4 °C.

### 2.2. Plant Material and Environmental Samples

Tomato plants (*Solanum lycopersicum* L.), cultivar (cv.) Roma, susceptible to *R. solanacearum* but free of the pathogen, were grown 3–4 weeks in TEKU: PL3040/20 and vermicoco (cocopeat) 100% in jiffis at 20–25 °C during the daytime and 18–20 °C during the nighttime, under greenhouse conditions at the Vegetal Production Unit of the Central Service for Experimental Research (SCSIE) at the Universitat de València (UV). Plant extracts were prepared by crushing 0.5 g of tomato plant stems in 4.5 mL of Phosphate Buffered Saline (PBS) at 10 mM, pH 7.2, and then filtering the extract to prepare *R. solanacearum*-spiked samples, as indicated in Section 2.3.

Soil samples from a tomato field and river water from *R. solanacearum*-free areas in the southeast of Spain were used to prepare *R. solanacearum*-spiked samples, as indicated in Section 2.3.

### 2.3. Bacterial and Phage Dilutions, and DNA Purification

Suspensions of 10^9^ PFU/mL of the mix of the three phages and 10^9^ CFU/mL of *R. solanacearum* were performed in PBS at 10 mM, pH 7.2, separately. Serial dilutions of 10^8^ to 1 PFU/mL of the phages and 10^8^ to 1 CFU/mL of the bacterium were performed in PBS and in river water, with extract of tomato plants cv. Roma and soil from pathogen-free samples. For direct amplification (without DNA purification), each dilution performed above was further diluted 100-fold in PBS. Total DNA was extracted from each dilution of all samples using the cetyltrimethyl ammonium bromide (CTAB) method [45]. DNA concentration and quality were determined spectrophotometrically at 260 nm and 280 nm with a Nanodrop (ND-2000, Thermo Fisher, Wilmington, DE, USA).

### 2.4. Design of Primers and Probes

To design appropriate primers and probes, the nucleotide sequences deposited in GenBank of complete genomes of the phages vRsoP-WF2 (MN685189), vRsoP-WM2 (MN685190), and vRsoP-WR2 (MN685191) were used. Alignments of the nucleotide sequences of the phages were carried out using Blast tools from the National Center for Biotechnology Information (NCBI) and Geneious Prime v2023.0.1 software. Primer Express software (Applied Biosystems, Waltham, MA, USA) was used to obtain the sequences of specific primers and TaqMan probes of *R. solanacearum* phages. In addition, primers and TaqMan probes specific for phage vRsoP-WR2 were designed based on the insertion of 480 bp present in their nucleotide sequence [28].

The primers B2-I-F/B2-II-R and the TaqMan probe B2-P specific to strains of *R. solanacearum* Phylotype IIB-1 (race 3, biovar 2) were described by Fegan et al. [46] and Weller et al. [35] and recommended in the EPPO Standard for *R. solanacearum* detection [47] (Table 1). The TaqMan probe of *R. solanacearum* was labeled in 5′ with fluorescent dye HEX and 3′ with quencher (IABkFQ).

### 2.5. Quantitative Duplex PCR

TaqMan assays for quantitative real-time PCR (qPCR) were performed in the StepOne Plus Sequence Detection System (Applied Biosystems) at the Genomics Unit of the SCSIE at the UV. The individual reaction cocktail in a final volume of 10 μL contained 1X TaqMan Universal PCR Master Mix (Applied Biosystems), 1 μM of each primer, 150 nM of TaqMan probe, and 2 μL of sample. The duplex reaction cocktail contained 1X TaqMan Universal PCR Master Mix (Applied Biosystems), 1 μM of each primer for the phages and for the bacterium, 150 nM of TaqMan probe for the phages and for the bacterium, and 2 μL of sample. Real-time PCR amplification consisted of one step at 95 °C for 10 min followed by 40 cycles of amplification (95 °C for 15 s and 60 °C for 1 min). Data acquisition and analysis were performed with the StepOne software v2.3. The default threshold set by the machine was slightly adjusted above the noise to the linear part of the growth curve, at its narrowest point, according to the StepOne manufacturers (Applied Biosystems).

### 2.6. Quantitative Multiplex PCR

TaqMan assays for multiplex qPCR were performed in a QuantStudio 5 Real-time PCR System (Applied Biosystems) at the Central Unit for Research in Medicine (UCIM) at the UV. The reaction cocktail in a final volume of 10 μL contained 1X TaqMan Universal PCR Master Mix (Applied Biosystems), 0.75 μM of each primer for phage vRsoP-WR2, 0.75 μM of each universal primer for the three phages, 0.75 μM of each primer for the bacterium, 150 nM of each TaqMan probe, and 2 μL of sample. Real-time multiplex PCR amplification consisted of one step at 95 °C for 10 min followed by 40 cycles of amplification (95 °C for 15 s and 60 °C for 1 min). Data acquisition and analysis were performed with QuantStudio Design and Analysis software v1.5.1. The default threshold set by the machine was slightly adjusted above the noise to the linear part of the growth curve, at its narrowest point according to the manufacturers (Applied Biosystems). To determine the theoretical sensitivity and the reliability of the multiplex real-time PCR for each sample type, three replicates of each assay were undertaken using the ten-fold serial dilutions of the single phage and the single bacterium, and the phage plus bacterium mixture.

**Figure 1:**
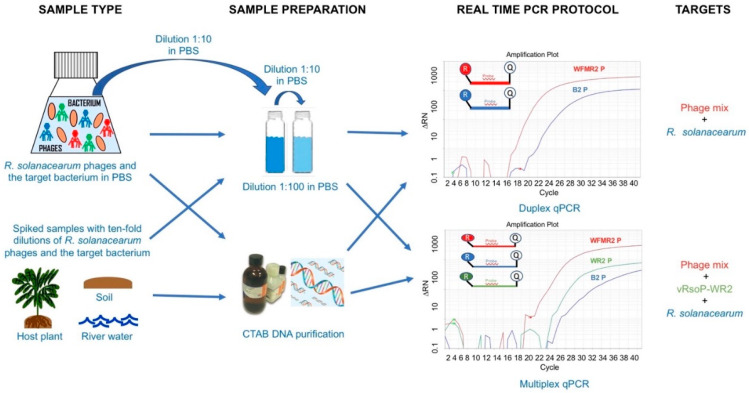
Graphical summary of the methodology developed for the detection and quantification of the *Ralstonia solanacearum* phages vRsoP-WF2, vRsoP-WM2, and vRsoP-WR2, and the target bacterium in environmental water, soil, and plant material using duplex and multiplex qPCR.

A summarization of the development of the TaqMan qPCR protocols for specific DNA detection and quantification of *R. solanacearum* and the phages vRsoP-WF2, vRsoP-WM2, and vRsoP-WR2, and their applications, is represented in Figure 1.

## 3. Results

### 3.1. Specificity of PCR of R. solanacearum Phages

**Figure 2:**
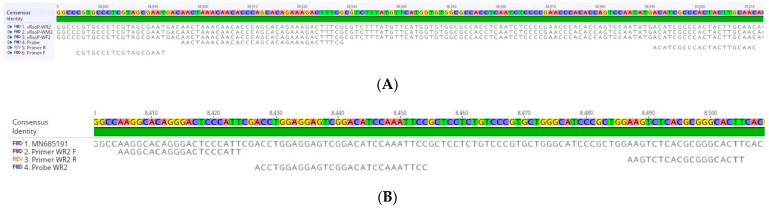
Nucleotide alignment of the sequences of *Ralstonia solanacearum* phages vRsoP-WF2, vRsoP-WM2, and vRsoP-WR2 in Geneious Prime software. Primer and probe sequences of the three phages (**A**), and primer and probe sequences specific to phage vRsoP-WR2 (**B**).

The primers WFMR2 F, WFMR2 R, and TaqMan probe WFMR2 P (Table 1; Figure 2A) were designed in ORF 52–53 (phage terminase small subunit) [28] and were able to specifically amplify the three *R. solanacearum* phages tested, vRsoP-WF2, vRsoP-WM2, and vRsoP-WR2. No amplification was obtained from *R. solanacearum* suspensions, nor from pathogen-free samples of river water, tomato plants, and soil used as controls. The primers WR2 F, WR2 R, and TaqMan probe WR2 P (Table 1; Figure 2B) were designed in ORF 17 (phage-restriction endonuclease) [28] and were able to specifically amplify the vRsoP-WR2 *R. solanacearum* phage. No amplification was obtained from phages vRsoP-WF2 and vRsoP-WM2, *R. solanacearum* suspensions, nor from pathogen-free samples of river water, tomato plants, and soil used as controls. In vitro assays confirmed the previous in silico specificity analysis performed with the sequences of the phages retrieved from databases.

### 3.2. qPCR Standard Curves for Quantification of R. solanacearum Phages and Bacterium

**Figure 3:**
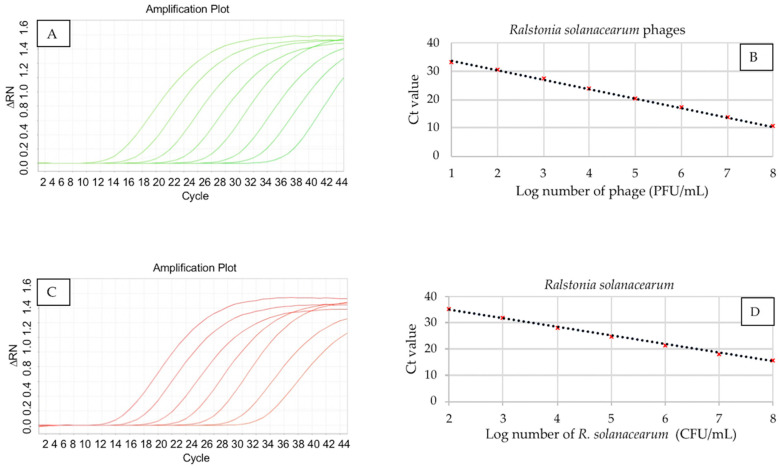
Quantification range of real-time duplex PCR. Amplification curves of 10-fold serial dilutions from 10^8^ to 10 PFU/mL for vRsoP-WF2, vRsoP-WM2, and vRsoP-WR2 phages (**A**), and standard curve (**B**). Amplification curves of 10-fold serial dilutions from 10^8^ to 10^2^ CFU/mL for *Ralstonia solanacearum* (**C**), and standard curve (**D**). Standard curves were obtained with mean values of three replicates for each of the ten-fold serial dilutions.

The quantification range of duplex PCR was established from 10^8^ to 10 PFU/mL of *R. solanacearum* phages and from 10^8^ to 10^2^ CFU/mL of *R. solanacearum* due to the reliability of three replicates (Figure 3).

The Ct value for the standard curve of the phages vRsoP-WF2, vRsoP-WM2, and vRsoP-WR2 ranged from 10.42 to 33.07 for 10^8^ to 10 PFU, respectively. The mathematical equation that represents the curve is: y = 3.321x + 36.916, with a correlation coefficient (R^2^) value of 0.9987. For quantitation of vRsoP-WR2 phage particles only, with the cocktail of duplex PCR, the same mathematical coefficients were obtained as when using a mixture of particles from all three phages. The Ct value for the *R. solanacearum* standard curve ranged from 15.84 to 35.20 for 10^8^ to 10^2^ CFU, respectively. The mathematical equation that represents the curve is: y = 3.291x + 41.529, with a correlation coefficient (R^2^) value of 0.9973. The Ct value for each dilution was the mean of three repetitions.

### 3.3. Sensitivity of Multiplex qPCR in the Detection of R. solanacearum Phages and the Bacterium in Different Types of Samples

A comparison of the sensitivities achieved by multiplex real-time PCR in the detection of the three phages and the bacterium using different types of samples with DNA purification or without it (direct samples) is shown in Table 2.

For the phage quantification, the detection limit was 10 PFU/mL in PBS buffer, 10^2^ in river water and tomato plant extracts, and 10^3^ PFU/g in soil. For the *R. solanacearum* quantification, the detection limit was 10^2^ CFU/mL in PBS, 10^3^ in river water and in tomato plant extracts, and 10^4^ PFU/g in soil. When different methods of sample preparation were compared, direct methods yielded similar or identical results as when a laborious DNA purification protocol was used. The phage- and/or bacterium-free samples and PCR cocktail controls were negative by all tested protocols.

**Figure 4:**
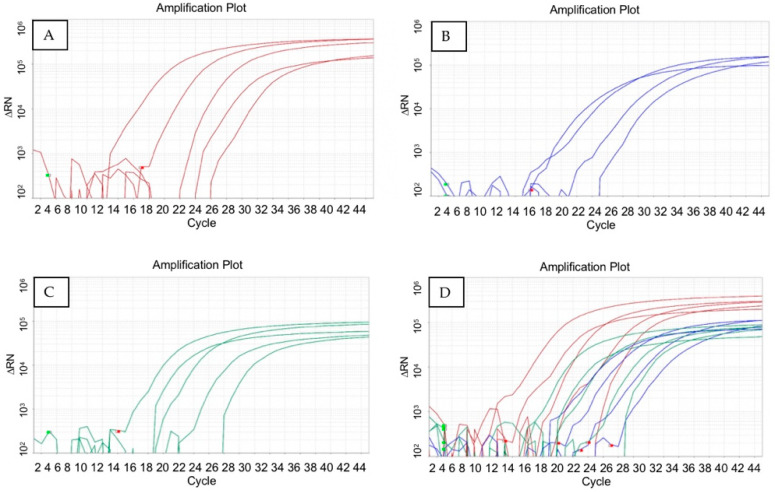
Real-time multiplex PCR. Amplification curves of serial dilutions from 10^6^ to 10^2^ PFU/mL of vRsoP-WF2, vRsoP-WM2, and vRsoP-WR2 phages ((**A**)—red), amplification curves of serial dilutions from 10^6^ to 10^3^ CFU/mL of *Ralstonia solanacearum* ((**B**)—blue), amplification curves of serial dilutions from 10^6^ to 10^3^ CFU/mL of vRsoP-WR2 phage ((**C**)—green), and amplification curves of serial dilutions from 10^6^ to 10^2^ CFU/mL of vRsoP-WF2, vRsoP-WM2, and vRsoP-WR2 phages, and from 10^6^ to 10^3^ CFU/mL of bacteria ((**D**)—red + green + blue). Dilutions of phages and the bacterium were performed in river water and direct sample preparation was used. Amplifications were performed in triplicate.

The sensitivity of the multiplex qPCR was the same as each individual amplification for vRsoP-WR2 phage, the three *R. solanacearum* phages, and the bacterium (Table 2). No differences on curve behavior were observed when vRsoP-WF2, vRsoP-WM2, and vRsoP-WR2 phages (Figure 4A), *R. solanacearum* (Figure 4B), or vRsoP-WR2 phage (Figure 4C) were amplified individually or all together (Figure 4D), showing no interference between the different primes and TaqMan probes used. Similarly, no differences in the efficiency and curve behavior were observed when different types of samples (river water, tomato plant extracts, or soil) were used.

## 4. Discussion

Bacterial wilt caused by *R. solanacearum* is one of the most important bacterial diseases concerning solanaceous plants and ornamentals of economic importance worldwide. Outbreaks in the field have frequently been associated with detection of the bacterium in watercourses, where it can survive for long periods as a free-living form and/or in roots of semi-aquatic weeds or other reservoir plants, or in the soil. In the host, detection of the pathogen can be hindered by the occurrence of latent or symptomless infections. Bacterial wilt management often fails due to the limited efficacy and environmental impact of agrochemical methods, and the lack of resistant cultivars. *R. solanacearum* is therefore subjected to strict regulation and included in international quarantine procedures [12,47]. Biological control methods are being explored. Among them, three lytic phages, vRsoP-WF2, vRsoP-WM2, and vRsoP-WR2, were isolated and further characterized because of their successful biocontrol of the pathogen [23,24,25,26,27]. As they proved to be specific for *R. solanacearum*, detection of at least one of them in environmental samples could be useful for the development of a sensitive, indirect, phage-based detection of the pathogen, since phage concentrations are usually ten times higher than those of their bacterial target [48]. Further, simultaneous sensitive detection and accurate identification of bacterium and phages are important to deepen knowledge on their interaction in the environment for improvement of the prevention and management of the disease.

There is a number of molecular tests that have been validated and can be routinely used for detection and identification of *R. solanacearum* (former phylotype II of the *Ralstonia solanacearum* species complex) and the phylogenetically related species *R. pseudosolanacearum* (former phylotypes I and III) and *Ralstonia syzygii* subsp. *indonesiensis* (former phylotype IV of the complex) for screening of plant material and other environmental samples, and some of them are also quantitative [47]. Concerning *R. solanacearum*, real-time qPCR protocols have been reported either based on SYBR green detection or TaqMan probes [35,36,37,38,39,40,41], and the design of specific primers and DNA probes from the 16S rDNA region [35,40] or the 16–23S rRNA spacer sequence [39]. *R. solanacearum* has also been detected from soil and plant tissues by qPCR based on the integration of the rapid self-replicating ability of phages [49]. However, all these methods have been developed to target the pathogenic bacterium, even when phages have been used in the protocol. There is no previous report on the combined or simultaneous detection of *R. solanacearum* and several specific phages able to lyse it.

In this work, specific primers and TaqMan probes were designed and evaluated for simultaneous detection and quantification of *R. solanacearum* and the phages vRsoP-WF2, vRsoP-WM2, and vRsoP-WR2, by duplex and multiplex qPCR in water, soil, and plant material. The genomic sequences of the three phages were compared with sequences obtained from public databases to identify regions with differences. As the sequences of vRsoP-WF2 and vRsoP-WM2 are nearly identical [28], it was not possible to design a TaqMan probe to differentiate between them. In contrast, vRsoP-WR2 has an insert of 480 bp between positions 8619 and 9095 compared to the other two phages [28]. Therefore, primers and a specific TaqMan probe could be designed only for vRsoP-WR2 in ORF 17 (putative endonuclease). *In silico* and *in vitro* analyses showed that the sequence of the designed primers and the TaqMan probe were specific for vRsoP-WR2. Additionally, primers and a TaqMan probe for the universal detection of the three phages were designed in the ORFs 52–53 (phage terminase small subunit) and validated. *In silico* analysis showed that the designed universal phage primers and probe also amplified the related *Ralstonia* sp. phages RsoP1EGY and DU_RP_I. However, amplification with other *Ralstonia*-related phages, such as PSG 11 and PSG11.1 [28], is not expected due to sequence homology.

The amplification protocols for vRsoP-WR2 individual qPCR and the qPCR for the three *R. solanacearum* phages were optimized, as well as the multiplex qPCR for vRsoP-WR2, the three phages, and the bacterium. Amplification curves were obtained from all the positive samples, without amplification of the negative controls or DNA from other phages and/or other bacterial species present in plant extracts, soil, and irrigation water samples. No interference was observed between probes and fluorophores.

The sensitivity of real-time qPCR for the detection of the three *R. solanacearum* phages together (universal), the vRsoP-WR2 phage, *R. solanacearum*, and the multiplex (phages and bacterium) protocol, was determined in environmental water, plant material, and soil. With respect to the phages, sensitivities of detection in river water were appropriate to the concentrations reported for *R. solanacearum* phages, which were estimated between 10^2^ and >10^3^ lytic viral particles/mL in natural river water samples [30]. Similar sensitivities were obtained in samples of more complex composition, such as tomato plant extracts and soil, where the presence of PCR inhibitors has been commonly described [35,37,50]. This is particularly significant in soil samples, where inhibitors such as humic acids and the microbial diversity in their culturable and unculturable states cause the difficulty of detection of a specific microorganism [37]. With respect to *R. solanacearum*, sensitivity of detection in PBS without previous DNA purification was in agreement with 10^2^ cells/mL obtained using the same primers and TaqMan probe by Weller et al. from cells suspended in water [35]. This sensitivity in water would not allow for a successful detection of *R. solanacearum* in river water in cases where natural population numbers of the bacterium have been reported to range from 10 to 80 CFU/mL [16]. The same authors obtained less sensitivity of *R. solanacearum* detection from potato extracts, pointing out the presence of qPCR inhibitors, and suggesting the need for the use of pathogen enrichment procedures [35]. Thus, the analytical sensitivity established in international procedures for extracts of potato heel-end cores spiked with *R. solanacearum* is 10^5^ CFU/mL using the B2-I-F/B2-II-R/B2-P assay [35,47]. Since the protocols developed in this work for the simultaneous detection of the phages and bacterium were about one order of magnitude more sensitive in detecting the phages than the target bacterium, they could be successfully applied for a highly sensitive detection of *R. solanacearum* as indicators of the pathogen contamination in different types of plant and environmental samples.

A direct method of sample preparation was compared and validated for use in real-time qPCR. Conventional PCR-based techniques require purified total DNA or RNA as templates in amplification reactions. However, nucleic acid purification steps are laborious and expensive, limiting the number of samples that can be processed. In real-time PCR protocols, especially when a TaqMan probe is used, nucleic acid purifications can be partially or totally circumvented using direct sample preparation methods such as dilution or spot immobilization on membranes [51,52,53,54,55,56,57,58,59]. Data obtained in this work showed that the dilution method of sample preparation allowed for the same sensitivity as conventional DNA extraction protocols. The dilution method requires fewer steps, is less labor-intensive and faster, and is more cost-effective than traditional purification DNA methods, favoring high-throughput results.

The qPCR protocols developed in this work are appropriate for analytical purposes, with excellent limits of detection and quantification. Phage isolation is generally carried out by plaque assays, with the limitations of their identification based on their lytic activity on indicator bacterial hosts and the need for relatively high numbers of phage particles in the sample [32]. Therefore, these molecular protocols for combined detection of *R. solanacearum* and the phages vRsoP-WF2, vRsoP-WM2, and vRsoP-WR2 substantially improved traditional methods, allowing for the sensitive, precise, reliable, and quick detection and quantification of phages and bacteria in tomato plant material, soil, and irrigation water, even without previous DNA purification, saving time and reagents by reducing the cost of the screening tests.

## Figures and Tables

**Table 1:** Sequences of primers and probes for *Ralstonia solanacearum* phages vRsoP-WF2, vRsoP-WM2, and vRsoP-WR2, and the bacterial species *R. solanacearum*.

Name	Sequence (5′–3′)	Reference
WFMR2 F	CGTGCCCTCGTAGCGAAT	This work
WFMR2 R	GTTGCAAGTAGTGGGCGATGT	This work
WFMR2 P	FAM-AACTAAACAACACCCAGCACAGAAAGACTTTCG-TAMRA	This work
WR2 F	AAGGCACAGGGACTCCCATT	This work
WR2 R	AAGTGCCCGCGTGAGACTT	This work
WR2 P	Cy5-ACCTGGAGG/TAO/AGTCGGACATCCAAATTCC-RQ	This work
B2-I-F	TGGCGCACTGCACTCAAC	[35,46]
B2-II-R	AATCACATGCAATTCGCCTACG	[35,46]
B2-P	HEX–TTCAAGCCGAACACCTGCTGCAAG-IABkFQ	[35]

**Table 2:** Sensitivity of PCR protocols in the detection of *Ralstonia solanacearum* phages vRsoP-WF2, vRsoP-WM2, and vRsoP-WR2, and *R. solanacearum* in PBS, river water, tomato plant extracts, and soil from pathogen-free samples, with and without DNA purification.

Real-Time PCR Protocol	Types of Samples						
	PBS	River Water		Tomato Plant		Soil	
	Direct ^a^	Direct	DNA ^b^	Direct	DNA	Direct	DNA
*R. solanacearum* phage cocktail (PFU/mL) (vRsoP-WF2, vRsoP-WM2, vRsoP-WR2)	10	10^2^	10^2^	10^2^	10^2^	10^3^	10^3^
vRsoP-WR2 phage (PFU/mL)	10	10^2^	10^2^	10^2^	10^2^	10^2^	10^2^
*R. solanacearum* (CFU/mL)	10^2^	10^3^	10^4^	10^3^	10^3^	10^4^	10^4^
Multiplex:							
*R. solanacearum* phage cocktail (PFU/mL) (vRsoP-WF2, vRsoP-WM2, vRsoP-WR2)	10	10^2^	10^2^	10^2^	10^2^	10^2^	10^2^
vRsoP-WR2 phage (PFU/mL)	10	10^2^	10^2^	10^2^	10^2^	10^3^	10^3^
*R. solanacearum* (CFU/mL)	10^2^	10^3^	10^4^	10^3^	10^3^	10^4^	10^4^

^a^ Without DNA purification. ^b^ CTAB DNA purification.

## Data Availability

Data are contained within the article.
